# Concentration of dioxin and screening level ecotoxicity of pore water from bottom sediments in relation to organic carbon contents

**DOI:** 10.1007/s10646-020-02318-w

**Published:** 2020-12-06

**Authors:** Agnieszka Baran, Magdalena Urbaniak, Magdalena Szara, Marek Tarnawski

**Affiliations:** 1 Department of Agricultural and Environmental Chemistry, University of Agriculture in Krakow, Al. Mickiewicza 21, 31-120 Krakow, Poland; 2 European Regional Centre for Ecohydrology of the Polish Academy of Sciences, Tylna 3, 90-364 Lodz, Poland; 3 Department of Hydraulic Engineering and Geotechnics, University of Agriculture in Krakow, Al. Mickiewicza 24/28, 30-059 Krakow, Poland

**Keywords:** Pore water, PCDDs/PCDFs, DOC, Ecotoxicity, Biotests

## Abstract

The information about concentrations of dioxin in pore water, ecotoxicity and DOC and TOC content can be key factor for the prediction of the fate of dioxins in the aquatic environment as well as an ecological risk assessment. The aims of the study were to assess the concentration of PCDDs/PCDFs and ecotoxicity of pore water and to compare above results in relation to the dissolved organic carbon (DOC) and total organic carbon (TOC) content. The concentration of dioxins was assessed using an enzyme-linked immunoassay test, while the ecotoxicity of pore water was determined using a crustacean *Daphnia magna* and bacteria *Aliivibrio fischeri*. The studies were conducted on two different dammed reservoirs Rożnów (catchment basin of an agricultural character) and Rybnik (catchment basin of an industrial character) located in southern Poland. The concentration of dioxins in pore water was between 8.56 to 90.92 ng EQ/L, with a significantly higher concentration in the pore water from the Rożnów Reservoir than the Rybnik Reservoir. The DOC content in pore water was from 30.29 to 63.02 mg/L (Rożnów Reservoir) and from 35.46 to 60.53 mg/L (Rybnik Reservoir). Higher toxic responses were recorded for *A. fischeri* than for *D. magna*. Moreover a significantly higher toxicity for both tested organisms was indicated in pore water from the Rożnów Reservoir. Besides of TOC and DOC, the fine fractions of the sediments were particularly important in the concentration of dioxin in pore water. The other pore water parameters, such as pH and EC can influence the toxicity of water for organisms. The result indicate complex relationships between the PCDD/F, ecotoxicity and DOC, TOC concentration in pore water and confirms that these parameters are important in terms of water environmental contamination.

## Introduction

Among the contaminants of the aquatic environment, a significant role is played by persistent organic pollutants (POPs), such as polychlorinated dibenzo-*p*-dioxins (PCDDs), polychlorinated dibenzofurans (PCDFs) (Ying et al. 2009; Förstner et al. 2016; Roumak et al. 2018; Urbaniak et al. 2016). PCDDs/PCDFs toxicity, persistence and bioaccumulation are widely recognised as posing a risk to living organisms (Nie et al. 2013; Kukučka et al. 2015). Bottom sediments are considered to be an important place for the accumulation of dioxins. Dioxins are characterised by high hydrophobicity; therefore, in water ecosystems, they quickly bound to organic carbon fractions in the suspended phase and in this form are transferred to sediments (Urbaniak et al. 2015; Louchouarn et al. 2018). Sediment pore water is defined as the water occupying the space between sediment particles, which constantly remains in contact with sediments; therefore, bottom sediments and pore water may exchange pollutants (Simpson and Batley 2016). It is known that the pore water test is more likely to detect the toxicity of substances than the solid-phase tests (Roig et al. 2011; Buruaem et al. 2013). Pore water can be an important exposure route for benthic organisms, because there is equilibrium between the phases adsorbed in the organic matter, carbon and the dissolved forms. This makes pore water a valuable tool for the assessment of the mobility, bioavailability and toxicity of different inorganic and organic contaminants (Ying et al. 2009; Roig et al. 2011; de Castro-Català et al. 2016). Dissolved organic carbon (DOC) in pore water plays an important role, e.g. it provides flux of sedimentary organic carbon to the overlying water, preserves organic matter, and it is also responsible for the transport and bioavailability of contaminants (O’Loughin and Chin et al. 2004; Akkanen et al. 2005; Ripszam et al. 2015; Fox et al. 2018; Dong et al. 2019). Pore water DOC is generally composed of the high-molecular-weight fraction, which consists of humic substances (Burdige 2001; O’Loughin and Chin et al. 2004). Several studies have demonstrated that humic substances can bind many inorganic and organic compounds (Bai et al. 2018; Baran et al. 2019a). Al-Reasi et al. (2012) have also observed that DOC reduces Cu toxicity to the *Daphnia magna*. In an aquatic environment, binding of Hg and MeHg to DOC can decrease the bioavailability of both forms of Hg to phytoplankton (Gorski et al. 2008; Chen et al. 2014). Akkanen et al. (2005) reported the influence of DOC on the bioavailability of hydrophobic organic substances; the authors also showed that increased binding led to decreased bioavailability.

The persistent organic pollutants present in the pore-water can be a measure of potentially mobile and bioavailable fraction (Wiberg et al. 2009; Simpson and Batley 2016; Niehus et al. 2018). The relation between freely dissolved concentrations of dioxin, ecotoxicity and content of DOC in the pore-water and TOC content in bottom sediments is useful for the prediction of the fate of dioxins in the environment as well as an ecological risk assessment (Frankki et al. 2007; Persson et al. 2008).

The aims of the present study were to 1) assess the concentration of PCDDs/PCDFs in pore water from two dam reservoirs, 2) assess the ecotoxicity of pore water, and 3) compare the obtained results in relation to the DOC and TOC content. The concentration of PCDDs/PCDFs was assessed using an enzyme-linked immunoassay test, while the ecotoxicity of pore water was investigated using two organisms-*Daphnia magna* (Daphtoxkit) and *Aliivibrio fischeri* (Microtox).

## Material and methods

### Study sites and sample processing

**Fig. 1:**
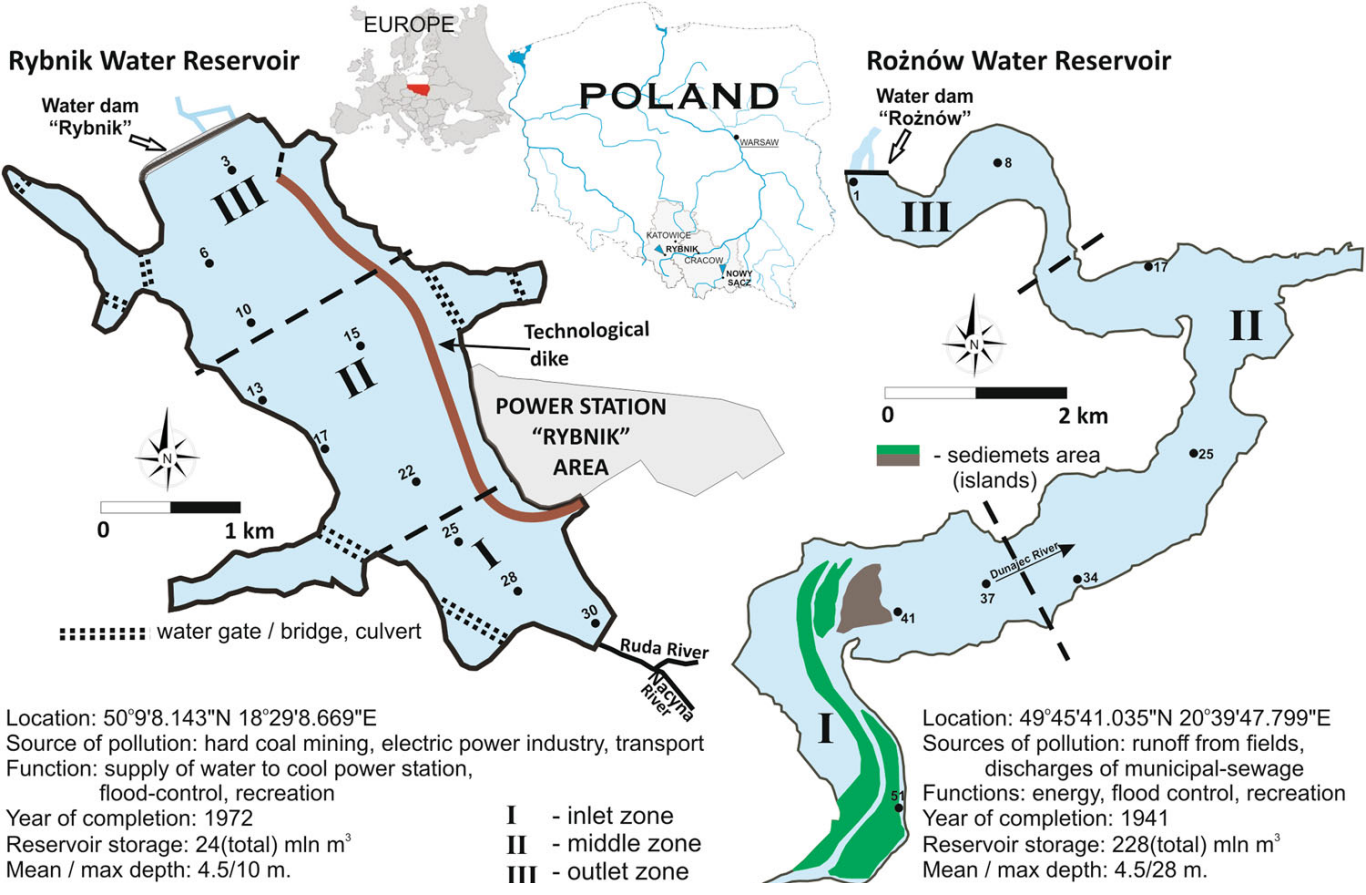
Localization of reservoirs and sampling points

The studies were conducted on two dammed reservoirs: Rożnów (the Dunajec River) and Rybnik (the Ruda River). The test subjects are two dammed reservoirs of varying characters located in southern Poland (Fig. 1). The Rożnów Reservoir has been functioning for almost 80 years and it encloses a mountainous catchment basin of an agricultural character, which is intensely subjected to the process of intense siltation (Baran et al. 2019b). The second subject—the Rybnik Reservoir—is a reservoir subjected to a strong human impact, as it constitutes the production process of a coal power plant. The reservoir constitutes a part of the technological chain of the Rybnik power plant as an essential source of cooling water as well as direct receiving water of treated industrial sewage (Baran et al. 2019a). It encloses a small catchment area, which is highly urbanised and strongly influenced by heavy industry (Baran et al. 2019a). In our previous studies, the concentration of PCDDs/Fs in the sediments form Rybnik reservoir ranged from 1.65 to 32.68 pg TEQ g^−1^ (Baran et al. 2020). Morover, we found that PCDD/F concentrations in the sediments were 2–38-fold higher than the Sediment Quality Guidelines limit, indicating high ecological risk potential. The source of PCDDs/Fs in the bottom sediments from the Rybnik reservoir were from combustion processes, transport, wastewater discharge, high-temperature processes and thermal electricity generation (Baran et al. 2020). Inflow of inorganic and organic pollution into the Roznów reservoir is high due to soil erosion, insufficient municipal wastewater treatment and village wastewaters (lack of sewage system in villages around the reservoir) and are emitted from fossil fuel (coal) combustion Morover, the silting process contributes to the inflow of fine fractions of both natural and anthropogenic origin is important source of pollutions in the bottom sediment (Baran et al. 2019b).

The samples of sediments were collected using an Ekman sampler from 18 set locations (8 from the Rożnów Reservoir and 10 from the Rybnik Reservoir). The samples were collected in each sampling point of the upper layer of bottom sediments (0–15 cm). The sediments were placed inside polyethylene containers and transported to the laboratory. Pore water samples were obtained from wet sediments in 200 ml centrifuge tubes, by centrifugation at 3000 rpm for 30 min. (Simpson and Batley 2016). The samples of pore water were placed in 50 cm^3^ glass conical test tubes and refrigerated in a fridge in the dark at 4 °C for later analyses.

### Chemical analysis and ecotoxicity bioassay

#### Basic chemical

The following properties of pore water have been determined: dissolved organic carbon (DOC), pH and electrolytic conductivity (EC). pH of pore water samples were determined by potentiometric method (pH – meter CP - 401, ELMETRON, Poland) and EC by conductometric method (conductivity/oxygen meter CCO - 401, ELMETRON, Poland). In order to analyse DOC, the samples of pore water were filtered through a 0.45 μm membrane filter (Akkanen et al. 2005). The content of DOC was assessed using TOC analyser 1200 (Thermo Elektron). In bottom sediments the total organic carbon (TOC) content was analysed using a CNS analyser (Vario EL Cube, Elementar Analysensysteme 2013) (Baran et al. 2019a, b, c).

#### PCDDs/PCDFs determination using Enzyme-Linked Immunosorbent Assay

The assays used in the study were purchased from Abraxis LLC (Warminster, USA). The Abraxis Dioxin/Furan ELISA is an indirect enzyme-linked immunosorbent assay (ELISA) for the screening of PCDDs/PCDFs in water, soil and sediment samples. The antibody used in Abraxis Dioxin/Furan ELISA is capable of binding to the majority of toxic congeners of PCDDs/PCDFs characterised by their Toxicity Equivalence Factors (TEFs) (Tuomisto et al. 2006; Van den Berg et al. 2006) however, it has also cross-reactivity with other less-toxic PCDD/PCDF congeners. Consequently, the used bioassay is a suitable tool to measure the total PCDDs/PCDFs toxicity (named as ELISA-EQ) of a given sample, without differentiating individual PCDD/PCDF congeners.

A detailed analytical procedure was described in our earlier study by Urbaniak et al. (2016) and Kobusińska et al. (2020). Briefly, a 125 μL of each calibration standard (0, 2.5, 5, 10, 25 and 50 ng EQ/L), the same volume of positive control (3 ng EQ/L) and pore water were mixed with an (125 μL) equal volume of an antibody solution and incubated in a glass tube for 60 min. After incubation, an aliquot of 100 μL from each vial was transferred to an antigen-coated well in a 96-microwell plate, and again incubated for 60 min. The content of each well was decanted to remove the solution containing any unbound reagents and each well was washed four times using 1× washing buffer solution. After this step, an aliquot of 100 μL of enzyme conjugate solution was added to each well and incubated for 30 min. Following the incubation, the contents of the wells were again decanted, and each well was washed four times using 1× washing buffer solution. After the washing step, an aliquot of 100 μL of chromogenic enzyme substrate solution was added to each well and the plate was incubated for 20 min in the dark. In the final step, an aliquot of 100 μL of stop solution was added into each well. The absorbance was measured at 450 nm using a Labsystems Multiskan RC 351 spectrophotometer. The concentrations of PCDDs/PCDFs were determined using a standard curve and presented as ELISA-equivalencies (ELISA-EQs).

In order to achieve high analytical standards and control the correctness of the method, each analytical batch contained a sample blank, a control sample of known concentration (3 ng EQ/L), calibration standards and samples. Duplicate analyses were used to verify the precision and the test reproducibility was measured using the coefficient of variation (CVs) (according to the manufacture’s instruction, the analytical procedure has to be repeated if the CVs exceeded the value of 12% for calibration standards and 15% for the samples). The least detectable dose (LDD), estimated as 90%B/Bo, was 2.5 ng EQ/L; samples displaying a concentration lower than LDD were considered have the concentration equal to the half of the LDD value (1.25 ng/L).

#### Ecotoxicity bioassay

The acute ecotoxicity assessment of each pore water samples was examined using two screening biotests: Daphtoxkit F magna and Microtox. In the Daphtoxkit biotest, a crustacean was used: *Daphnia magna*, and in the Microtox bacteria: *Aliivibrio fischeri*. Both biotests are the most internationally used bioassays for monitoring the toxicity of waters (Mankiewicz-Boczek et al. 2008; de Castro-Català et al. 2016). *D. magna* is a major trophic component of aquatic food webs and it is an important biological indicator of aquatic pollution (Dai et al. 2013; Huang et al. 2017). Moreover, *D. magna* is a primary consumer, making the connection between producers and secondary consumers as small fish.

Crustacean ecotoxicity was evaluated by assessing the mortality of *Daphnia magna* after an incubating the tested organisms with analysed pore water samples for 24 h, in the dark, at 20 °C (Daphtoxkit 1996, Kyzioł-Komosińska et al. 2016). The Microtox test was used to determine the luminescence inhibition of *Aliivibrio fischeri* before and after 15 min incubation of the bacterial suspension with the pore water sample (Tarnawski and Baran 2018). The decrease in luminescence was measured with an 81.9% Screening Test using the Microtox M500 Analyser (MicrobicsCorporation 1992). The toxicity of samples was presented as a Percent Effect (PE%). The toxicity classification system was used to estimate pore water toxicity: PE% lower than 20%, it indicated a lack of a significant toxic effect; 20% ≤PE < 50%, the sample was considered to be of a low toxicity. Samples, where PE% was higher than 50%, were classified to be toxic (Persoon et al. 2003).

### Statistical analysis

The results were verified statistically using the Statistica 12 software package, and included the mean, standard deviation, minimum, maximum and the coefficient of variation (CV%). The differences between the means were analyzed by U Manna–Whitney’ a test. In all cases, the level of significance was set at *p* < 0.05. Principal Component Analysis (PCA) was used to determine the relationship between analysed parameter. PCA analysis was conducted using varimax rotation standard. As limiting criteria the number of factors used the scree test.

## Results

### Chemical parameters of pore water

**Fig. 2:**
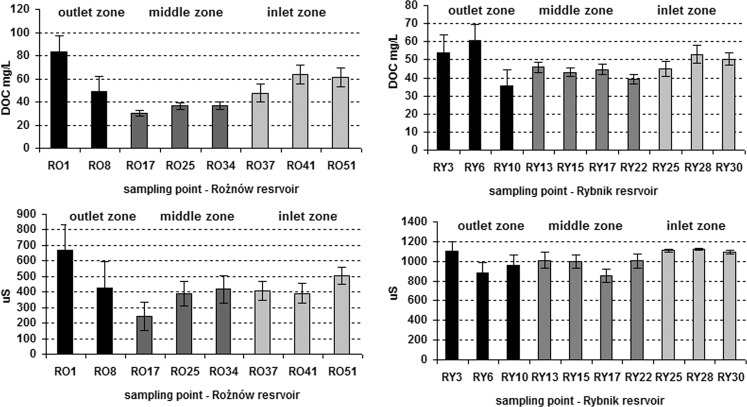
Comparison of DOC concentration and EC in the pore water from different sampling points in the reservoirs

The basic properties of pore water were shown in Table 1 and Fig. 2. The pore water was characterised by neutral and very slight alkaline reactions, and the pH ranged from 6.95 to 7.38 (Rożnów Reservoir) and from 7.04 to 7.58 (Rybnik Reservoir). The electrolytic conductivity ranged from 246 (RO17) to 1120 (RY28) μS. The pore water from the Rybnik Reservoir showed a significant (2.6-fold) higher EC value than pore water from the Rożnów Reservoir (Table 1). The highest value of EC was observed in pore water from the outlet zone (Rożnow Reservoir) and the inlet zone (Rybnik Reservoir) (Fig. 2). The studies showed a similar mean content of DOC in the pore waters of both reservoirs. The content of DOC in pore water within the reservoir was from 30.29 (RO17) to 63.02 (RO6) mg/L (Rożnów Reservoir) and from 35.46 (RY10) to 60.53 (RY6) mg/L (Rybnik Reservoir), respectively. However, a greater variation in the content of DOC was found in pore water from the Rożnów Reservoir (CV = 25%) and a lower variation was found in pore water from the Rybnik Reservoir (CV = 16%). In both reservoirs, the highest content of DOC in pore water was indicated in sampling points from the outlet zone, close to the dam, and the lowest was indicated in the middle part of reservoirs (Fig. 2). The TOC content in the bottom sediments differed significantly between the reservoirs. The TOC content was almost 5-fold higher in the bottom sediments of the Rybnik Reservoir compared to the bottom sediments of the Roznów Reservoir (Table 1). The analysis of the particle size fraction determined the dominance of the silt (67%± 18) and clay (20%±10) fraction in the bottom sediments of the Rożnów Reservoir (Szara et al. 2020). The bottom sediments coming from the Rybnik Reservoir contain from 48 to 99% sand and from 1 to 52% mud (silt + clay) (Baran et al. 2019a).

**Table 1:** Basic chemical properties of bottom sediment and pore water

Reservoir	Parameter	TOC%	DOC mg/l	pH	EC μS
Rożnów	Mean ± SD	1.75* ± 0.59	46.06 ± 11.48	7.17 ± 0.16	431.1* ± 119.4
	Range	0.65–2.51	30.29–63.02	6.95–7.38	246–668
	CV%	33	25	2	28
Rybnik	Mean ± SD	8.64* ± 4.82	47.13 ± 7.72	7.33 ± 0.18	1012* ± 94.3
	Range	0.22–12.93	35.46–60.53	7.04–7.58	852.7–1120
	CV%	56	16	3	9

*denote a significant difference at the *p* < 0.05, no asterisk—statistically non-significant results

**Fig. 3:**
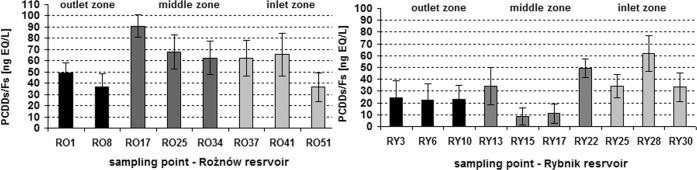
Comparison of dioxin concentration in the pore water from different sampling points in the reservoirs

The concentration of dioxins in pore water varied widely from 8.56 to 90.92 ng EQ/L depending on the sampling point and the reservoir, with a significantly higher concentration found in the pore water from the Rożnów Reservoir than the Rybnik Reservoir (Table 2, Fig. 3). The mean PCDDs/PCDFs concentration in pore water was 58.90 ng EQ/L for the Rożnów Reservoir and 30.19 ng EQ/L for the Rybnik Reservoir. However, the greatest variability in PCDDs/PCDFs concentration was observed in pore water from the Rybnik (CV = 54%) compared to pore water from the Rożnów Reservoir (CV = 31%). In the case of pore water from the Rożnów Reservoir, the lowest concentrations were observed in the outlet zone, close to the dam (points sampling RO1, RO8) and the highest in the middle part of the reservoir (points sampling RO17, RO25, RO34) (Fig. 3). The Rożnów Reservoir has been functioning for almost 80 years and it encloses a mountainous catchment basin of an agricultural character, which is intensely subjected to the process of intense siltation (Baran et al. 2019b). Pollution may come from also the reservoir’s own catchment area: tourist centers, local pollution of tourist sites as well as motor-boating and sailing harbors, as well as waste combustion. The higest content of dioxins in the central part of the reservoir may be due to the location of the marinas and motorboats in this area. In contrast, the results from the Rybnik Reservoir showed the highest concentration of PCDDs/PCDFs in pore water from the inlet zone, while the lowest was observed in the middle part of the reservoir in sampling points RY15 and RY17. The main sources of pollutants in the Rybnik reservoir are the metallurgical industry, combustion of coal and dry precipitation (Baran et al. 2017, 2019a). Moreover, pollution enter to inlet part of the reservoir, together with municipal wastewater, industrial sewage discharged by the Rybnik power plant, and long-range transport associated with the contaminated water of the Ruda river.

**Table 2:** Elisa–EQ concentration of PCDDs/PCDFs (ng EQ/L) in pore water and response of *D. magna* and *A. fischeri*

Parameter	Reservoir	Mean±SD	Range	CV%
PCDDs/PCDFs ng EQ/L	Rożnów	58.90* ± 18.04	36.44–90.92	31
	Rybnik	30.19* ± 16.32	8.56–61.80	54
*D. magna*	Rożnów	22 ± 36	0–100	167
	Rybnik	10 ± 24	0–75	242
*A. fischeri*	Rożnów	46* ± 15	14–60	33
	Rybnik	28* ± 17	−8 to 45	50

*denote a significant difference at the *p* < 0.05, no asterisk—statistically non-significant results

### Ecotoxicity of pore water

**Fig. 4:**
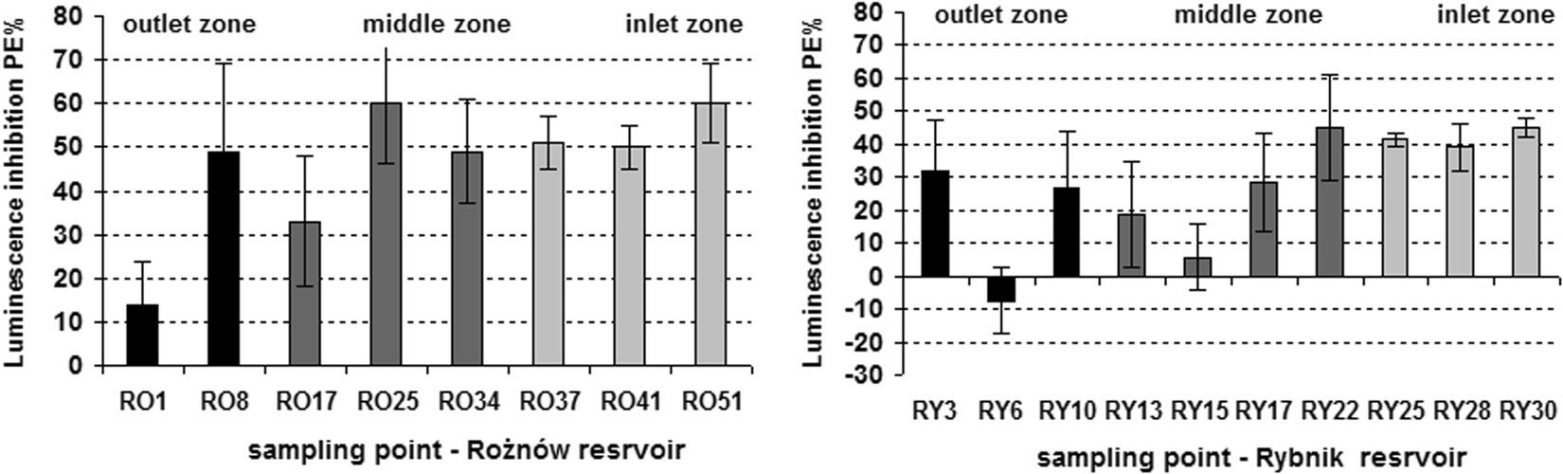
Comparison of *A. fischeri* luminescence inhibition in pore water from different sampling points in the reservoirs

Mortality of *D. magna* ranged from 0 to 100% (R25) (Rożnów Reservoir) and from 0 to 75% (R15) (Rybnik Reservoir) (Table 2). The toxic effect on *D. magna* was demonstrated only in 3 samples of the Rożnów Reservoir pore water (RO25, RO34, RO41) and 2 samples of the Rybnik Reservoir (RY15, RY17). *A. fischeri* luminescence inhibition was within 14 (RO1) to 60% (RO25, RO51, Rożnów Reservoir) and -8 (RY6) to 45% (RY 22, RY30, Rybnik Reservoir) (Table 2, Fig. 4). Generally, a significantly higher toxicity for both tested organisms was indicated in pore water from the Rożnów Reservoir, while a higher variability was observed for the Rybnik Reservoir. In both reservoirs, pore water from the middle zone showed the highest toxicity to the *D. magna* and from the inlet zone to the *A. fischeri* (Fig. 4). The mean ecotoxicity of the pore water samples for *A. fischeri* can be placed in the following order: inlet zone > middle zone > outlet zone (Fig. 4). In the Rożnów Reservoir, 50% of the pore water samples were toxic for *A. fischeri*, whereas only 25% of the samples were toxic for *D. magna*. Moreover, 36% (*A. fischeri*) and 13% (*D. magna*) of the samples were classified as slightly toxic. Most of the examined pore water samples (63%) from the Rożnów Reservoir were classified as non-toxic for *D*. magna while 13% of the pore water samples for *A. fischeri* were classified as non-toxic. In the case of pore water from the Rybnik Reservoir, 72% of the samples were classified as slightly toxic, and 30% as non-toxic for *A. fischeri*. In this study, there were no toxic samples (PE > 50%) of pore water for *A. fischeri*. Pore water samples collected from the Rybnik Reservoir were generally non-toxic (80%) for *D. magna*. A slightly toxic and a toxic effect for crustaceans was observed only in 10% of samples, respectively.

### PCA analyses

The Principal Components Analysis (PCA) indicated generally different effect of chemical properties on ecotoxicity of pore waters (Table 3). PCA allowed to extract two principal components (PCs), explaining the total variance of the dataset: 64.526 (Rożnów reservoir) and 67.641% (Rybnik Reservoir). For Rożnów reservoir, PC1 explaining 39.753% of the total variance had significant positive loadings on TOC, silt and PCDD/F concentrations in pore water and with negative loadings on DOC concentration and sand. PC2 explains 38.772% of the total variance, with a positive loading for the pH and response of *A. fischeri*, and negative loading on EC of pore water. In the pore water of Rybnik reservoir, the first component (PC1) accounted for 37.553% of the total variance, with a positive loading for TOC, DOC, EC and PCDD/F concentration, and negative loading with pH, *D. magna* response and sand (Table 3). PC2 explaining 30.088% of the total variance, with a positive loading for silt and clay, and negative for EC and *A fischeri* response. The combination of properties in PC1 (Rożnów Reservoir) suggested the positive relationship between TOC, silt content in sediment (positive) and DOC, sand (negative) and PCDD/F concentration in pore water. The significant loadings for *A. fischeri* in PC2 suggested the positive relationship with pH and negative relation with clay and EC (Rożnów Reservoir). In the pore water of the Rybnik reservoir the combination of date in PC1 showed the positive relationship between TOC, DOC, EC and PCDD/F concentration. The significant loadings for EC, silt, clay and *A. fischeri* suggested the relationship between these parameters in pore water (Rybnik Reservoir). Morover, the combination of properties in PC2 suggested the other factors affecting ecotoxicity of pore water for *D. magna*. Moreover, the opposite loads signs for *A. fischeri* and *D. magna* (PC1) in both pore water suggested different sensitivity both organism.

**Table 3:** Component matrix of variables

Variables	PCA 1	PCA 2	PCA 1	PCA 2
	Rożnów		Rybnik	
TOC	**0.717**	0.033	**0.614**	0.049
DOC	−**0.762**	0.171	**0.619**	0.466
pH	0.174	**0.537**	−**0.824**	0.452
EC	−0.457	−**0.674**	**0.527**	−**0.600**
*D. magna*	0.411	0.519	−**0.817**	−0.007
*A. fischeri*	−0.303	**0.871**	0.336	−**0.873**
PCDD/F	**0.770**	0.293	**0.638**	−0.414
sand	−0.932	0.154	−0.713	−0.288
silt	0.852	0.254	0.212	0.855
clay	0.386	−0.738	0.212	0.855
**Total variance %**	39.753	38.772	37.553	30.088
**Cumulative variance %**	39.753	64.526	37.553	67.641

Factor loadings exceeding 0.5 are shown in bold

## Discussion

It is worth emphasising that the properties of pore water depend mainly on the properties of bottom sediments from which the pore water was extracted. In the studies, pore water was extracted from two sets of bottom sediments with varying physical and chemical properties. In the pore water of the Rożnów Reservoir higher content of dioxins was demonstrated, along with increased ecotoxicity of pore water to the test organisms. We believe that such a situation might be caused by a lower content of total organic carbon in the bottom sediments of the Rożnów Reservoir (Table 1). According to the literature, the content of TOC plays a significant role in the distribution and sorption of dioxins and other POPs in the bottom sediment (Gao et al. 2016; Čonka et al. 2014). Sorption of PCDD to organic carbon and rapid partitioning to sediments might have reduced the bioavability of PCDDs directly from the water column (Servos et al. 1992). Organic matter in the solid phase increases the partitioning of organic pollution to the sediment, so it would be expected that dioxin partitioning to water increases with a lower TOC sediment content. Ankkanen et al. (2005), Klimkowicz-Pawlas et al. (2017) and Baran et al. (2019a) pointed that an important role in sorption and desorption processes of inorganic and organic pollutants is played by dissolved organic carbon (DOC) and black carbon (BC). Many authors found a strong positive relationship between the concentration of inorganic and organic pollutants as well as the DOC content in bottom sediments and pore water (Liu et al. 2008; Al-Reasi et al. 2012; Ripszam et al. 2015; Baran et al. 2019a). Akkanen et al. (2005) found that in natural water, DOC significantly decreased the bioavailability of benzo[a]piren, while the bioavailability of atrazine was not affected by DOC. Organic carbon (OC) in pore water is differentiated by size: particulate organic carbon (POC), and dissolved organic carbon (DOC) (Fiedler et al. 2008; Persson et al. 2008). DOC is commonly defined as organic matter in water samples smaller than 0.45μm, while POM is frequently defined as organic carbon larger than 0.7μm (Fiedler et al. 2008). Persson et al. (2008) found that chlorinated compounds (CPs, PCPPs, PCDEs and PCDD/Fs) due to the differences in their physicochemical properties vary in their partitioning between colloidal fractions and the filtered water. Frankki et al. (2007) studied the partitioning of CPs, PCDEs, PCDFs and PCDDs between dissolved (DOC) and particulate (POC) organic carbon, indicating that compounds have a weaker sorption to DOC and possibly a higher mobility compared to compounds associated to POC. The proportion of the bound fraction increased with an increasing hydrophobicity of the chlorinated compounds. In the pore water the colloidal organic carbon can play a significant role in the adsorption and transport of dioxin (Persson et al. 2008). Besides organic matter, the clay and silt fractions of sediments play an important role in the distribution of dioxins in bottom sediments (Zhang et al. 2010; Li et al. 2012). In our previous study, fine fractions of sediments were demonstrated to play a dominant role in the distribution of dioxins in Rybnik Reservoir (Baran et al. 2019c). Vácha et al. (2011) and Lee et al. (2006) also observed a poor relation between the concentration of organic pollutants and the content of TOC in the sediment. Moreover, Lee et al. (2006) suggested that the concentration of dioxins in silt and clay particles was up to 16 times higher than in sand or coarse. Small-sized particles have large specific areas and a high adsorption capacity of organic compounds (Junttila et al. 2015). Moreover, many aquatic organisms take up nutrition from the finest fraction through filtering. Therefore, fine particles (2-10 μm), which are consumable by water organisms, can be important in determining the bioavailability of dioxins (Lee et al. 2006). Dioxin concentrations in sediments equilibrate between solid phases of sediment, pore-water and overlying water. If dioxin concentrations in pore-water are higher than in the overlying water, one can expect a diffusive transport of dissolved dioxin from the sediment to the water column (Wiberg et al. 2009). The transfer of dioxin between sediment and water is also affected by particle deposition, resuspension and bioturbation. In the present study, PCA found positive (Rybnik reservoir) and negative (Rożnów reservoir) relation between the DOC and dioxin concentration in the pore waters. However, in both pore waters, the relationships between TOC in bottom sediments and dioxin concentrations in pore waters were positive, and the TOC content in the sediments of the Rybnik Reservoir was almost 5-fold higher then in the sediments form the Rożnów Reservoir. All of the above relationships may suggest their poor sorption by DOC and higher mobility of dioxin and thus ecotoxicity to test organisms in pore water form Rożnów Reservoir. Therefore, an indirect but key factor affecting the concentrations of dioxin in sediments can be an intense silting process of the Rożnów reservoir (Baran et al. 2019b). Ambiguous results indicate complex relationships between the parameters in pore water. In our studies, besides of TOC and DOC the silt and clay fractions of the sediments were particularly important in the concentration of dioxin in pore water. We think that the silt/clay fractions of bottom sediments play a role in the movement of dioxin, while the TOC and DOC fraction affects their sorption.

According to the literature, organisms have different sensitivities to various substances in bottom sediments and pore water (Hermann et al. 2016; Tarnawski and Baran 2018). In our studies also were indicated different sensitivities of the *A. fischeri* and *D. magna* for substances in pore waters (Table 2). In the studies, higher toxic responses were recorded in the Microtox than in the Daphtoxkit test. However, Lahr et al. (2003) observed that crustaceans were the most sensitive organisms in a battery of bioassays with pore water. Roig et al. (2011) and Kudłak et al. (2016) indicated that the algae test was more sensitive than other organisms in pore water and bottom sediment samples. In the studies of de Castro-Català et al. (2016), the following were used to asses the ecotoxicity of pore water: *A. fischeri, P. subcapitata and D. magna*, whereas the following were used for bottom sediments: *A. fischeri*, *C. riparius*. Similarly as in the presented studies, *A. fischeri* was an organism most sensitive to the studied pore water. The higher toxicity of the pore water for *A. fischeri* than for *D. magna* can be connected to the pathways of contaminant exposure. For bacteria, the main routes of substances are through diffusion of free dissolved chemicals (Tuikka et al. 2011). The main exposure route for *D. magna*, in turn, is contact with chemicals in the pore waters through a filter–feeding strategy (Kim et al. 2017). Pollutants bound to DOC are not bioavailable for fish and invertebrates (Ankkanen et al. 2005). Our earlier study has shown that the properties of DOC significantly affect the restriction of pollutant bioavailability (Baran et al. 2019a). The formed complexes between DOC and hydrophobic organic compounds are too big and too polar to diffuse through cell membranes of organisms for DOC to limit bioaccumulation of hydrophobic organic pollutants (Ankkanen et al. 2005). Servos et al. (1989) found that humic acid (DOC) reduced the apparent uptake rate constants of PCDDs by reducing the free water concentrations available to the fish. However, contaminants bound to DOC are easy uptake by bacteria and serve as a food source for bacteria-based food-webs (Ripszam et al. 2015). However in our studies, no significant relationship was found between the content of dioxins and DOC in the pore water and its toxicity to organisms. The results suggest that other factors influence the ecotoxicity of pore waters from both reservoirs. The higher EC in pore water in Rybnik may be indicating the presence of nutrients, metals or some pharmaceuticals which are ionized at pH around 7–7.5. In bottom sediments and pore water a range of substances are present starting from nutrients through organic compounds and inorganic contaminants. Each of them can have an impact on others in synergistic, antagonistic, additive or potentiation way. In the studies of Ying et al. 2009 and Baran et al. (2019a), it was also shown that organic carbon could not explain the observed toxicity patterns. Moreover, we think that lack of significant relation between DOC and toxicity of pore water was also connected with the procedures of preparing pore water samples for analysis. For ecotoxicity test, filtration of pore water samples was avoided. Filtered samples generally have lower toxicity than unfiltered samples, because filtration procedures remove a large proportion of fine or colloidal particles, which may be of importance in the toxicity assessment of pore water (Simpson and Batley 2016). For analysis of DOC pore water should be filtered. Ying et al. 2009 also indicated that hydrophobic compounds, like dioxins, were the main cause of the toxicity to *E. coli* in the pore water; however, these compounds were not responsible for the toxicity of pore water to luminescent bacteria. In pore water, toxicity could be also caused by sulphide, ammonia and nitrogen concentration (Rosen et al. 2008; Łukawska-Matuszewska et al. 2009). Studies of some authors prove a higher ecotoxicity of sediments compared to that of pore water (de Castro-Català et al., 2016; Tarnawski and Baran 2018). On the other hand, Roig et al. (2011), Buruaem et al. (2013) and Pini et al. (2015) concluded that studies of the ecotoxicity of pore water are a more sensitive indicator of negative changes within a water ecosystem than studies of the ecotoxicity of the solid phase of bottom sediments.

## Conclusion

To sum up, in the Rożnów reservoir higher content of dioxins was demonstrated, along with increased ecotoxicity of pore water to the organisms. Besides of TOC and DOC, the fine fractions of the sediments were particularly important in the concentration of dioxin in pore water. The pore water ecotoxicity wasn’t directly connected with to dioxin, DOC and TOC. However, the other pore water parameters, such as pH and EC, can influence the toxicity of water for test organisms. The results indicate complex relationships between the PCDD/F, ecotoxicity and DOC concentration in pore water and confirm that these parameters are important in terms of environmental contamination.
